# Introduction of Extract of Belladonna and Gummy Extract of Opium into the Urethra in Incarcerated Inguinal Hernia

**Published:** 1846-01

**Authors:** 


*Introduction of Extract of Belladonna and Gummy Extract of Opium into the Urethra in Incarcerated Inguinal Hernia.* By Dr. Tronsone.—The taxis, application of ice, venesections, clysters, &c., having all been employed in vain in a case of incarcerated hernia, in which the operation was obstinately refused, the author resorted to the method recommended by Riberi, by injecting into the urethra a mixture of ten grains of extract of belladonna and six grains of gummy extract of opium, by means of an elastic catheter, and applying some of the mixture to the hernia itself. Within a few hours after the injection the hernial swelling had disappeared, and all incarceration was removed.—*Medical Times* from *Schmidt's Jahrbuch.—Ibid.*

